# Preparation and Performance of PAN/PS/PMMA Ternary Blend-Modified Fiber Membranes via Centrifugal Spinning for Lithium-Ion Batteries

**DOI:** 10.3390/nano15110789

**Published:** 2025-05-24

**Authors:** Shunqi Mei, Feng Luo, Yi Xie, Bin Xu, Quan Zheng

**Affiliations:** 1 Hubei Digital Textile Equipment Key Laboratory, Wuhan Textile University, Wuhan 430073, China; sqmei@wtu.edu.cn (S.M.); 2215373064@mail.wtu.edu.cn (F.L.); 2415373048@wtu.edu.cn (Y.X.); 2315373009@mail.wtu.edu.cn (B.X.); 2 School of Mechanical & Electrical Engineering, Xi’an Polytechnic University, Xi’an 710048, China; 3 The Advanced Textile Technology Innovation Center (Jianhu Laboratory), Shaoxing 312000, China

**Keywords:** centrifugal spinning, fiber membranes, polystyrene, poly(methyl methacrylate), blending modification

## Abstract

Addressing the issues of poor thermal resistance in conventional polyolefin separators and the low production efficiency of electrospinning, this study innovatively employed high-efficiency centrifugal spinning technology to fabricate a ternary blended modified fiber membrane composed of polyacrylonitrile (PAN), polystyrene (PS), and polymethyl methacrylate (PMMA). By precisely adjusting the polymer ratio (8:2:2) and fine-tuning the spinning process parameters, a separator with a three-dimensional network structure was successfully produced. The research results indicate that the separator exhibited excellent overall performance, with a porosity of 75.87%, an electrolyte absorption rate of up to 346%, and a thermal shrinkage of less than 3% after 1 h at 150 °C, along with a tensile strength reaching 23.48 MPa. A lithium-ion battery assembled with this separator delivered an initial discharge capacity of 159 mAh/g at a 0.2 C rate and maintained a capacity retention of 98.11% after 25 cycles. Moreover, under current rates of 0.5, 1.0, and 2.0 C, the battery assembled with the ASM-14 configuration achieved high discharge capacities of 148, 136, and 116 mAh/g, respectively. This study offers a novel design strategy for modifying multi-component polymer battery separators.

## 1. Introduction

Lithium-ion batteries boast numerous advantages, including high energy density, excellent stability, and minimal side reactions. As a result, they have been widely investigated and applied in the automotive sector, portable devices, unmanned aerial vehicles, and utility-scale energy storage. The main components of a battery include the cathode, anode, separator, and electrolyte. The separator acts as a physical barrier between the battery electrodes and serves as a reliable bridge for ion transport, playing a crucial role in maintaining the battery’s sustainability [1,2,3].

At present, the mainstream commercial separators used in lithium-ion batteries (LIBs) are polyolefin membranes made from semicrystalline polymers such as polyethylene (PE) or polypropylene (PP), typically with a thickness of less than 25 μm. These separators achieve a microporous structure through a stretching process, in which the pores are formed by the phase separation between crystalline and amorphous regions. This type of structure is also referred to as a microporous membrane [4,5]. With the continuous advancement of lithium battery technology, the PP and PE separators produced using conventional dry and wet processes face inherent limitations in their material properties, such as low melting points and poor heat resistance, which make them prone to shrinkage at elevated temperatures. This shrinkage can lead to electrode short circuits during abnormal exothermic electrochemical reactions, thereby failing to meet the stringent safety requirements of lithium batteries [6,7].

Fiber membranes are typically fabricated using methods such as electrospinning, centrifugal spinning, and melt blowing. Owing to their interwoven network structure, they exhibit high porosity and a large specific surface area. Moreover, novel functional separators, such as ion-selective membranes and multifunctional composite separators integrated within the fiber membranes, have attracted considerable attention. Compared to traditional commercial microporous films, these advantages arise from the unique microstructure and design versatility of fiber membranes [8,9].

In recent years, electrospinning technology has gradually emerged as a key method for fabricating fiber membranes in the field of lithium-ion battery manufacturing, due to its unique processing advantages [10]. A wide range of polymers has been successfully electrospun into fiber membranes [11,12,13]. Pengqian Guo et al. [14] employed electrospinning to fabricate an alumina (Al_2_O_3_)/polyacrylonitrile (PAN) battery separator. The study demonstrated that this separator physically inhibits the growth of lithium dendrites, thereby enhancing the stability of the lithium interface and suppressing the shuttle effect of lithium polysulfides. Moreover, the battery assembled with the Al_2_O_3_/PAN separator exhibited excellent capacity and cycling stability. Xiu Shen et al. [15] employed an alumina coating technique to modify the surface of PVDF-HFP fiber materials produced via electrospinning, thereby developing a novel lithium battery separator. This material maintained a stable morphology even at a high temperature of 200 °C and exhibited excellent flame-retardant properties and electrochemical compatibility. Testing revealed that the enhanced separator not only improved electrolyte wettability and significantly boosted lithium-ion transport but also led to notable improvements in the battery’s charge–discharge performance and cycling stability. The analysis above indicates that fiber membranes produced via electrospinning possess advantages such as high porosity, excellent electrolyte absorption, and high ionic conductivity. However, this technique is hindered by low production efficiency—typically only 0.1–1 mL/h—which restricts its large-scale application as a lithium-ion battery separator [16,17].

Centrifugal spinning is a fiber fabrication technique in which the spinning solution is ejected from a spinning device during high-speed rotation. The rapid rotation generates centrifugal force that, once it exceeds the surface tension of the liquid phase, leads to the formation of liquid fibers. These jets undergo rapid solvent evaporation and stretching, resulting in the deposition of dry nanofibers onto the collector [18]. In terms of material compatibility, centrifugal spinning imposes no restrictions on polymer polarity, whereas electrospinning requires conductive solutions and relies heavily on polar solvents, thereby limiting material selection. Regarding fiber morphology control, centrifugal spinning produces fibers with better alignment, while electrospinning is prone to jet whipping, which leads to disordered fiber deposition—an issue that can adversely affect the tensile strength and thermal stability of the resulting fiber membranes. In contrast, centrifugal spinning offers a production rate that is two orders of magnitude higher than that of electrospinning, reaching 100–300 mL/h [19]. Moreover, it does not rely on high-voltage electric fields, making it inherently safer [20]. This fiber fabrication technique has been widely applied in fields such as biomedicine [21], energy [22], communications [23], and materials science [24]. Rihova et al. [25] were the first to utilize centrifugal spinning to fabricate biopolymer fibers blended with ZnO nanoparticles for the treatment of acne. Lai et al. [26] simulated the flow behavior of spinning solutions within various rotating nozzles and found that curved nozzles are more suitable for high-speed centrifugal spinning. Rihova et al. [27] employed centrifugal spinning to fabricate blended fibers composed of natural gum and polyethylene oxide, demonstrating potential applications in cosmetics and dermatology. Xia et al. [28] used centrifugal spinning to prepare lignin amine/cellulose acetate nanofibers, which exhibited high adsorption selectivity and are suitable for heavy metal ion adsorption.

To date, a wide range of materials have been employed for fiber fabrication via centrifugal spinning, including polyvinylidene fluoride (PVDF) [29], cellulose [30], polyvinylpyrrolidone (PVP) [31], polyacrylonitrile (PAN) [32], polyurethane (PU) [33], polystyrene (PS) [34], polylactic acid (PLA) [35], polyethylene oxide (PEO), and polyvinyl alcohol (PVA) [36]. Polyacrylonitrile (PAN) is commonly used as a matrix material for lithium battery separators due to its favorable properties, including high ionic conductivity, excellent electrolyte uptake, and outstanding thermal stability [37,38]. Studies have shown that the cyano groups (–CN) on the PAN backbone can interact with the carboxyl groups (–COOH) and Li^+^ ions in the electrolyte, thereby enhancing the compatibility between PAN and the electrolyte [39]. However, the cyano (–CN) groups can undergo passivation reactions with the lithium metal anode, adversely affecting the battery’s lifespan. In addition, PAN exhibits limited mechanical strength, which further deteriorates upon contact with plasticizers such as ethylene carbonate (EC) in the electrolyte. To address these shortcomings, researchers have adopted copolymerization or blending strategies to modify PAN and enhance its overall performance [40]. The benzene ring structure in the molecular chains of polystyrene (PS) endows the material with high tensile and compressive strength, as well as excellent resistance to cyclic stress and dimensional stability. In addition, PS also exhibits a high glass transition temperature and dielectric breakdown strength [41]. The molecular structure of poly(methyl methacrylate) (PMMA) is primarily composed of methyl and ester groups, which exhibit high chemical inertness and are unlikely to undergo side reactions with lithium metal. In addition, due to the similar polarity between its ester groups and carbonate-based electrolytes, PMMA shows excellent compatibility and strong affinity with electrolytes [42].

In summary, blending PAN, PS, and PMMA can further enhance the properties of pure PAN membranes, offering the potential to obtain fiber membranes with improved porosity, electrolyte uptake, mechanical strength, thermal stability, and overall battery performance. Moreover, employing the more efficient centrifugal spinning technique for membrane fabrication represents a promising direction for research. The ratio of PAN, PS, and PMMA is closely related to the spinnability and performance of the resulting fibers, making the determination of an optimal PAN/PS/PMMA spinning formulation critically important.

This study investigated the fabrication process of PAN, PAN/PS, and PAN/PS/PMMA blended nanofiber membranes using centrifugal spinning technology. The physical properties of these membranes were characterized, and their performance as separators in battery assemblies was evaluated.

## 2. Materials and Methods

### 2.1. Materials

The primary materials used in this study included polyacrylonitrile (PAN, Mw = 150,000), polystyrene (PS, Mw = 280,000), and polymethyl methacrylate (PMMA, Mw = 150,000), all purchased from Shanghai Aladdin Biochemical Technology Co., Ltd., Shanghai, China. N,N-dimethylformamide (DMF, analytical grade) was obtained from Sinopharm Chemical Reagent Co., Ltd., Shanghai, China. Acetylene black, polyvinylidene fluoride (PVDF), lithium metal, N-methyl-2-pyrrolidone (NMP), and lithium hexafluorophosphate (LiPF_6_) electrolytes were sourced from Dongguan Kelude Experimental Equipment Technology Co., Ltd., Dongguan, China. Celgard 2400 was purchased from Celgard LLC, Charlotte, NC, USA. All materials were commercially available and used as received without further purification. They were vacuum-dried at 60 °C for 12 h prior to use.

### 2.2. Preparation of PAN/PS/PMMA Ternary Blended Modified Fibrous Membranes

**Figure 1:**
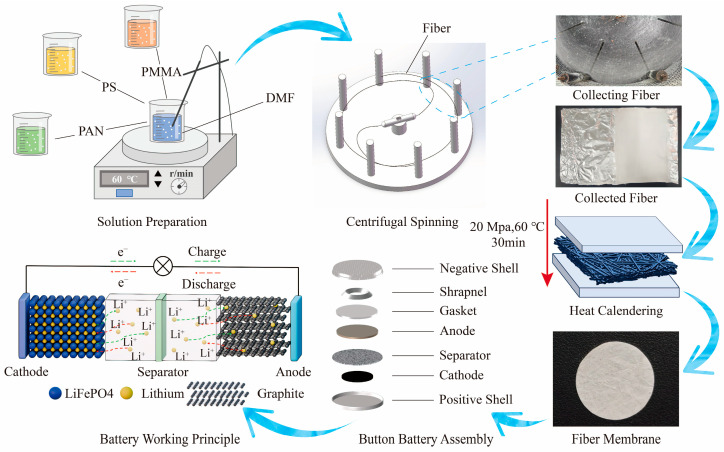
Preparation of fiber membranes using the centrifugal spinning technique and battery assembly.

The preparation process for the centrifugal spinning fiber membrane comprised several steps including solution formulation, spinning, fiber collection, fiber stacking, and thermal pressing, as illustrated in Figure 1. Certain amounts of PAN, PS, and PMMA were accurately weighed and added to a beaker. An appropriate amount of N,N-dimethylformamide (DMF) was then introduced as the solvent, and the components were mixed according to the desired mass fraction ratio. The beaker was placed on a magnetic stirrer and heated while stirring for a specific period to obtain a homogeneously blended spinning solution.

The relative humidity was maintained between 20% and 35%. At room temperature, fiber membranes were prepared using a custom-built centrifugal spinning apparatus. The collection distance was set to 20 cm. After spinning for 1 min, the process was halted. Fibers were subsequently sectioned and aligned along the fiber axis for layer-by-layer collection. This collection process was repeated four to five times. The assembled fibers were sandwiched between two stainless steel plates, each measuring 15 × 15 cm^2^, and then thermally pressed at 25 MPa and 60 °C for 30 min. Thickness measurements using a micrometer indicated that the fiber membrane achieved a final thickness of 50 ± 10 μm. After heat pressing, the fiber membrane was cut into circular discs and assembled into batteries.

During the spinning process, increasing the rotational speed enhances the jet stretching rate and solvent evaporation rate, thereby enabling the formation of finer fibers. A smaller needle aperture helps restrict the flow rate of the solution and reduces the initial jet radius. This increases the surface area-to-volume ratio of the jet, allowing the solvent to evaporate more rapidly and resulting in the formation of finer fibers. During the centrifugal spinning process for fiber fabrication, the mass concentration of the solution is directly related to its viscosity, serving as a decisive factor in determining the morphology of the resulting fibers. When the viscosity of the spinning solution is too low, its rheological properties lead to instability in the fiber-formation process, resulting in interruptions in the continuous jet stream and the formation of bead defects. Conversely, if the viscosity is too high, it restricts fiber stretching, causing an increase in fiber diameter or even preventing fiber formation entirely [43]. To investigate the effect of different polymer concentrations on the centrifugal spinning performance, PAN solutions with varying mass fractions were first used to carry out centrifugal spinning. The experimental conditions are outlined in Table 1.

Based on the optimized polymer concentration obtained from the formulations in Table 1, the preparation of PAN/PS/PMMA fiber membranes with varying blend ratios was subsequently investigated. The detailed experimental design is presented in Table 2.

In the experiments outlined in Table 2, the separators were designated as ASM-1, ASM-2, ASM-3, …, ASM-17. The fiber membranes prepared in this study, along with the commercial Celgard 2400 separator, were subjected to performance evaluation. The effects of varying PMMA and PS contents on the properties of PAN-based separators were systematically analyzed.

### 2.3. Microscopic Morphology and Performance Characterization

The microstructure of the fiber membranes was characterized using scanning electron microscopy (SEM, GeminiSEM 300, ZEISS, Oberkochen, Germany). Flat-surfaced fiber membrane samples were cut, sputter-coated with gold for 45 s, and subjected to SEM and energy-dispersive spectroscopy (EDS) analysis. For each fiber sample, 12 SEM images were captured, and the diameters of approximately 100 individual fibers were measured using ImageJ (Windows, ImageJ bundled with 64-bit Java 8) software.

The thermal stability of the separators was evaluated using thermogravimetric analysis (TGA, STA 2500 Regulus, NETZSCH, Selb, Germany). The mass change behavior of the separators was analyzed over a temperature range of 20 to 800 °C, with a heating rate of 10 °C/min.

Differential scanning calorimetry (DSC, 204F1, NETZSCH, Selb, Germany) was employed to evaluate the thermal stability of the separators by analyzing their thermal transitions. The measurements were conducted over a temperature range of 20 to 450 °C, using the same heating rate as in the TGA test.

The crystalline phases of the membranes were analyzed using an X-ray diffractometer (XRD, SmartLab SE, Rigaku, Tokyo, Japan) over a 2θ range of 10° to 60°.

The separators were cut into specimens with dimensions of 10 × 50 mm^2^ and an average thickness of 0.05 mm. Tensile tests were conducted using a universal testing machine (68TM-10, INSTRON, Norwood, MA, USA) to obtain the stress–strain curves. The stretching speed was set to 10 mm/min with a gauge length of 20 mm. Tensile strength was calculated based on the maximum force measured during the test, and the elongation at break was determined from the deformation length. The tensile strength was calculated using the following equation:

(1) $$\sigma =\frac{F_{max}}{A}$$

where σ represents the tensile strength; *F_max_* denotes the maximum force recorded prior to sample fracture; *A* is the cross-sectional area of the sample.

The elongation at break was calculated according to the following equation:

(2) $$Elongation\;at\;break\;(\%)=\frac{Lf}{L0}\times 100\%$$

where *Lf* is the deformed length, and *L*0 is the initial length.

The separators were placed in an oven and heated at 150 °C for 1 h to evaluate their thermal dimensional stability. The shrinkage ratio was calculated using the following equation:

(3) $$Thermal\;Shrinkage\;(\%)=\frac{Da-Db}{Da}\times 100\%$$

where Da and Db are the areas of the separator before and after heating at a specified temperature, respectively.

The hydrophilicity of the separator was measured using a contact angle goniometer (DSA30S, KRUSS, Hamburg, Germany). The separator was immersed in n-butanol solution for 1 h, and its porosity PPP was determined using the n-butanol absorption method. The porosity was calculated according to the following equation:

(4) $$\mathrm{Porosity}\;(\%)=\frac{W1-W0}{\rho 0\times V}\times 100\%$$

where *W*0 and *W*1 represent the mass of the separator before and after n-butanol absorption (g), respectively; *ρ*0 is the density of n-butanol (0.8098 g/cm^3^); *V* is the volume of the separator (cm^3^).

The electrolyte uptake of the separator was determined using the weighing method, measuring its mass before and after soaking in LiPF_6_ electrolyte for 1 h, as follows:

(5) $$\mathrm{Electrolyte}\;\mathrm{Uptake}\;(\%)=\frac{Mb-Ma}{Ma}\times 100\%$$

where *Ma* and *Mb* are the masses of the separator before and after electrolyte absorption, respectively.

### 2.4. Battery Performance

In this study, lithium-ion batteries were assembled in an ultra-high-purity glove box using a LiFePO_4_ cathode, lithium metal as the counter electrode, electrolyte, and the prepared separators. The charge–discharge performance, cycling stability, and rate capability of the cells were evaluated using a Neware battery testing system (CT-3008, Neware Electronics Co., Ltd., Shenzhen, China).

## 3. Results and Discussion

### 3.1. Microstructure, Elemental Distribution, and X-Ray Diffraction of the Membranes

According to the spinning experiment conducted based on the scheme in Table 1, the results indicated that the membrane morphology was optimal when the solution mass fraction was 18%. Therefore, in the subsequent experimental schemes, the solution mass fraction was designed to be 18%. Spinning experiments were performed for different ratios of PAN/PS/PMMA according to the scheme in Table 2. The results showed that the membranes had good morphology when the solution ratios were PAN/PS/PMMA = 10:0:0,8:2:0, 7:3:0, and 8:2:2, corresponding to ASM-1, ASM-3, ASM-6, and ASM-14, respectively.

**Figure 2:**
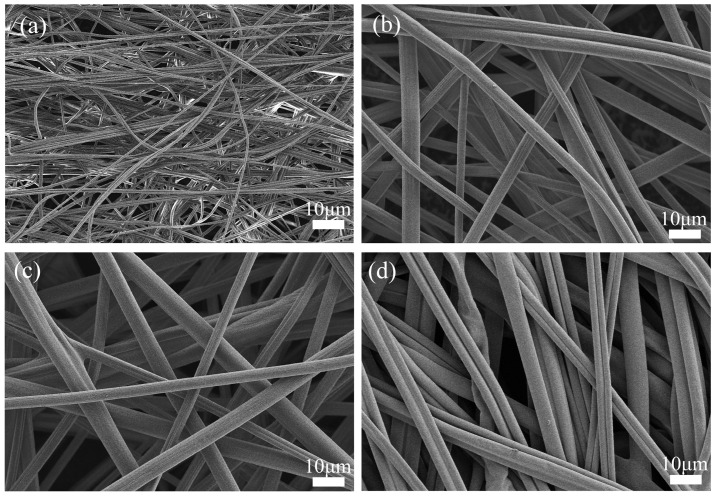
SEM images of the fiber membranes: (**a**) ASM-1; (**b**) ASM-3; (**c**) ASM-6; (**d**) ASM-14.

The microscopic morphology of the membranes is shown in Figure 2. The fibers in ASM-1 (Figure 2a) are entangled and relatively disordered. In contrast, the fibers in ASM-3 (Figure 2b) and ASM-6 (Figure 2c) exhibit a more uniform arrangement compared to ASM-1. ASM-14 (Figure 2d) displays highly oriented fibers, which are favorable for enhancing the axial strength of the membrane. The PAN/PS/PMMA fibers prepared via centrifugal spinning form a three-dimensional network structure, which is beneficial for improving ionic conductivity [44]. During the thermal pressing process, fiber junctions become bonded at their intersections. At these bonded points, fiber mobility is restricted, reducing the tendency for slippage, which in turn promotes a more uniform stress distribution and ultimately enhances the overall strength of the fiber membrane [45].

**Figure 3:**
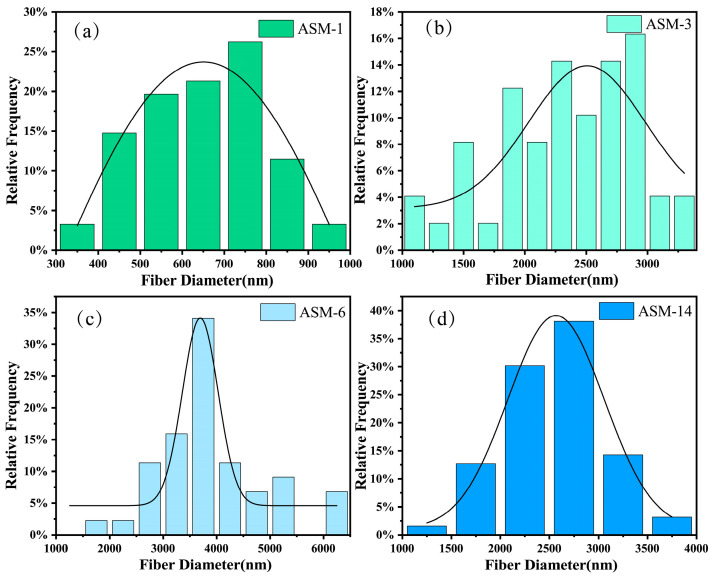
Fiber diameter distribution of the PAN/PS/PMMA membranes: (**a**) ASM-1; (**b**) ASM-3; (**c**) ASM-6; (**d**) ASM-14.

Figure 3 presents the fiber diameter distributions of the PAN/PS/PMMA membranes. The average diameters of ASM-1, ASM-3, ASM-6, and ASM-14 were 640, 2347, 4133, and 2576 nm, respectively. The fiber diameter of membranes prepared via centrifugal spinning is influenced by various factors, including the viscosity and concentration of the spinning solution, motor speed, and the configuration of the nozzle. In this study, the solution concentration, motor speed, and nozzle structure were kept constant; therefore, the solution viscosity was the primary factor affecting the fiber diameter. After the addition of PS, the rheological properties of the solution changed, leading to an increase in the viscosity of the spinning solution. This higher viscosity hindered the thinning of the spinning jet, resulting in larger fiber diameters. Therefore, within a certain range, the average fiber diameter increased with the PS content [46]. Another possible reason is that PAN and PMMA contain polar functional groups, while PS is a non-polar polymer. During solvent evaporation in the blended solution, phase separation may occur between the polar and non-polar polymers, which also contributes to the increase in fiber diameter. After the addition of PMMA, the diameter dispersion coefficient decreased, indicating that the incorporation of PMMA effectively improved the uniformity of the fibers. This may be attributed to the compatibilizing effect of PMMA, which stabilizes fiber formation by suppressing the phase-separation process.

**Figure 4:**
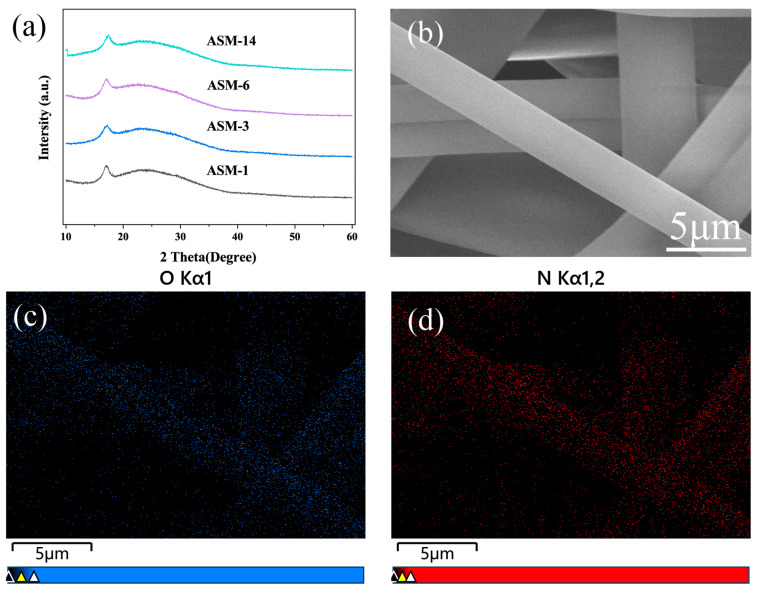
X-ray diffraction and EDS mapping of the PAN/PS/PMMA fibers: (**a**) XRD pattern; (**b**) SEM image; (**c**) oxygen-element mapping; (**d**) nitrogen element mapping.

Figure 4a presents the XRD patterns of the PAN/PS/PMMA fiber membranes. A strong diffraction peak appeared near 17°, corresponding to the characteristic diffraction of PAN, while a weaker diffraction peak was observed around 24°, which is attributed to the carbon peak of PAN [47,48]. After the incorporation of PS and PMMA, no significant shift in diffraction peaks or notable change in peak intensity was observed. This indicates that the blending did not lead to an increase in the crystallinity of the membranes, reflecting good compatibility among PAN, PS, and PMMA.

The EDS maps in Figure 4b–d demonstrate the uniform distribution of PAN and PMMA within the blended fibers, as indicated by the even spatial distribution of nitrogen (N) and oxygen (O) elements, with no evidence of local aggregation.

### 3.2. Contact Angle, Porosity, Electrolyte Uptake, and Tensile Strength

**Figure 5:**
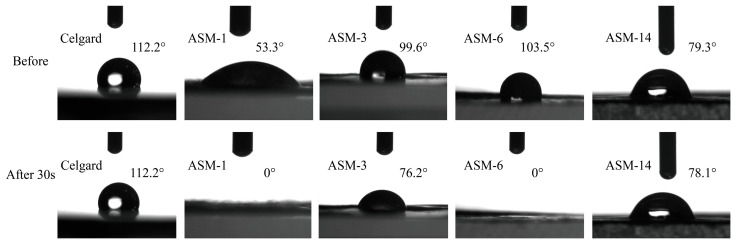
Initial and 30-s contact angles of the separators.

Figure 5 shows the initial contact angles and the contact angles after 30 ss for the PAN/PS/PMMA fiber membranes and the commercial Celgard separator. The initial contact angle of the commercial Celgard separator was measured to be 112.2°. The initial contact angles of ASM-1, ASM-3, ASM-6, and ASM-14 were 53.3°, 99.6°, 103.5°, and 79.3°, respectively. Among them, ASM-3 and ASM-6 exhibited relatively weak hydrophilicity, which can be attributed to the intrinsic hydrophobic nature of polystyrene (PS) [49]. Furthermore, the larger fiber diameters of ASM-3 and ASM-6 compared to those of ASM-1 also contributed to their reduced hydrophilicity. Due to the abundant porosity inherent in the PAN/PS/PMMA separators fabricated via centrifugal spinning, these membranes exhibited a certain degree of delayed water absorption. After standing for 30 s, the liquid droplets on ASM-1 and ASM-6 were completely absorbed into the membrane, and the contact angles of ASM-3 and ASM-14 also decreased. As a result, the difference in liquid uptake capacity between the ASM fiber membranes and the Celgard separator became more pronounced. With the incorporation of PMMA, ASM-14 exhibited enhanced hydrophilicity, which was beneficial for electrolyte uptake.

**Figure 6:**
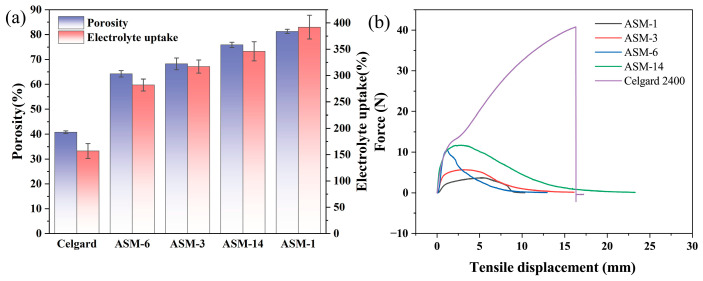
Electrolyte uptake and mechanical properties of the separator (**a**) porosity and electrolyte absorption rate of the membrane; (**b**) tensile strength of the membrane.

The porosity of the separators was measured using the n-butanol uptake method. The commercial Celgard separator exhibited a relatively low porosity of only 40.82%, whereas ASM-1 showed a significantly higher porosity of 81.27% (Figure 6a). With increasing PS content, the porosities of ASM-3 and ASM-6 decreased to 68.23% and 64.21%, respectively, indicating that a higher PS content leads to reduced porosity. This indicates that, within a certain range, increasing the PS content leads to a decrease in the porosity of the separator, primarily due to the significant increase in fiber diameter. However, all separators prepared via the centrifugal spinning method exhibited higher porosity than the commercial Celgard 2400 separator. This also suggests that separators fabricated via centrifugal spinning can absorb more electrolyte, which facilitates faster ion transport. Furthermore, with the addition of PMMA, the porosity of ASM-14 increased to 75.87%, enabling greater electrolyte uptake. This observation is consistent with the findings reported by Yanilmaz [42].

Enhancing the electrolyte uptake of the separator contributes to the optimization of lithium-ion battery performance. As shown in Figure 6a, the electrolyte absorption capacities of the Celgard separator and the PAN/PS/PMMA fibrous membranes were compared. Among them, ASM-1 exhibited the highest electrolyte uptake, reaching 392%. This is attributed to the presence of nitrile groups in PAN, which can coordinate with lithium ions, thereby improving the separator’s ability to dissolve Li^+^ and retain more electrolyte. The hydrophobic nature of PS led to a reduction in the electrolyte uptake of ASM-3 and ASM-6 to 317% and 282%, respectively. After PMMA was incorporated into the blend, the electrolyte uptake of the ASM-14 separator reached 346%, which is 2.2 times that of Celgard 2400 (157%). This enhancement is attributed to the ester functional groups in PMMA, which improve the wettability of the separator with the electrolytes.

Figure 6b presents the stress–strain curves of the PAN/PS/PMMA fiber membranes and the Celgard separator. According to Equation (2), ASM-14 exhibited an excellent elongation at break of 115%, compared to 81.5% for the commercial Celgard 2400 separator. This enhancement is attributed to the superior mechanical flexibility of the fiber membranes. The maximum tensile force sustained by the Celgard 2400 membrane was 40.79 N with a thickness of 0.025 mm, corresponding to a tensile strength of 163.16 MPa. In comparison, the maximum tensile forces for ASM-1, ASM-3, ASM-6, and ASM-14 were 3.76, 5.72, 10.48, and 11.74 N, respectively, corresponding to tensile strengths of 7.52, 11.44, 20.96, and 23.48 MPa. This indicates that the tensile strength of the PAN/PS/PMMA separator meets the mechanical strength requirement of 6.89 MPa for lithium battery separators [50]. The variation in tensile strength among ASM-1, ASM-3, ASM-6, and ASM-14 demonstrates that increasing the proportion of PS and PMMA in the blend solution enhances the tensile strength of fiber membranes. A higher PS content results in thicker fiber diameters, while an increased PMMA content improves the fiber alignment and produces more uniform diameters. The synergistic effect of PS and PMMA contributes to an overall improvement in membrane strength. The mechanical strength of the ASM membranes was significantly lower than that of the Celgard 2400 membrane. This disparity arises from the fact that Celgard 2400 is fabricated from polypropylene (PP) using a dry stretching process, which produces a highly oriented semi-crystalline lamellar structure. PP exhibited a high degree of molecular chain regularity, and the stretching process substantially increased its crystallinity, thereby imparting high tensile strength to the separator. In contrast, the centrifugal-spun fiber membranes in this study were composed of PAN, PS, and PMMA, which have significantly lower crystallinity than PP, resulting in comparatively inferior mechanical properties.

### 3.3. Thermal Stability and Dimensional Thermal Stability

**Figure 7:**
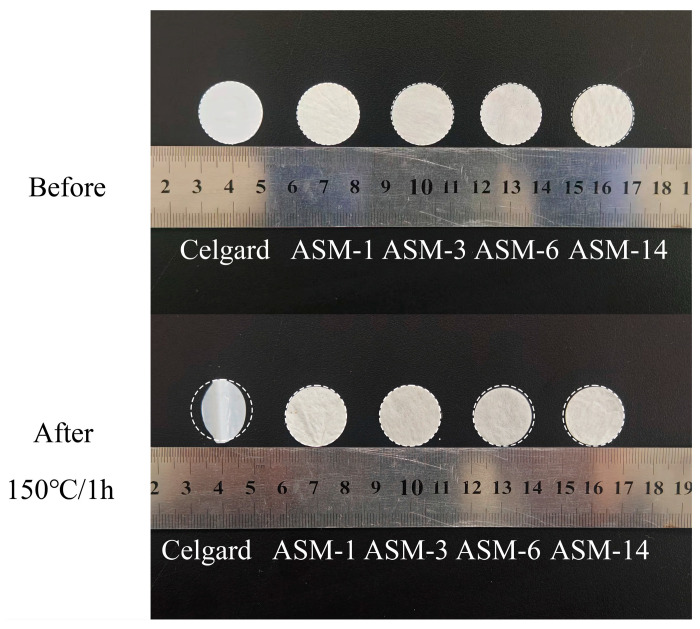
Comparison of the membranes before and after heating at 150 °C for 1 h.

The thermal stability and thermal dimensional stability of the separator are closely related to the safety of lithium-ion batteries. Figure 7 shows the thermal shrinkage behavior of these membranes after being subjected to 1 h at 150 °C. The commercial Celgard membrane exhibited significant thermal shrinkage, with a shrinkage rate of 28.9%. In contrast, the ASM-1, ASM-3, ASM-6, and ASM-14 membranes maintained their size well, with shrinkage rates of 0.28%, 0.12%, 3.18%, and 2.85%, respectively. This is attributed to the excellent thermal stability of the PAN, PS, and PMMA materials.

**Figure 8:**
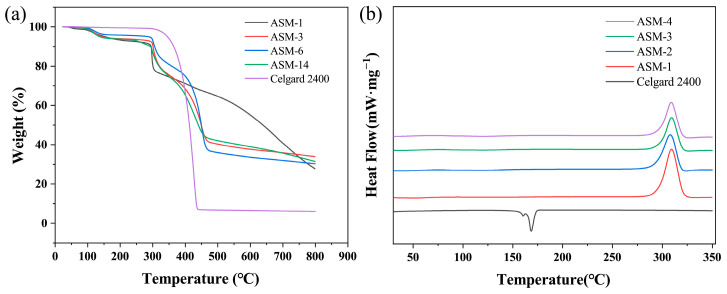
Thermal stability of the separator: (**a**) TGA curves of the PAN/PS/PMMA fiber membrane and Celgard 2400; (**b**) DSC curves the of PAN/PS/PMMA fiber membrane and Celgard 2400.

The thermal decomposition and carbon residue rates of the PAN/PS/PMMA fiber membrane and commercial Celgard separator were investigated in the temperature range of 20–800 °C. The TGA curves are shown in Figure 8a. All ASM membranes exhibited a glass transition. The commercial Celgard 2400 membrane showed significant thermal decomposition around 300 °C, and it was completely decomposed around 440 °C. At 800 °C, the residual mass retention was less than 7%. In contrast, the thermal weight loss range of the PAN/PS/PMMA fiber membranes was between 290 and 460 °C. The weight loss of ASM-1 occurred most rapidly between 290 °C and 300 °C, with the accelerated decomposition rate attributed to the cyclization reaction of the nitrile groups in PAN [51]. The weight loss of ASM-3 and ASM-6 primarily occurred in two stages: 290–300 and 380–460 °C. In the first stage, the accelerated decomposition rate was attributed to the cyclization reaction of the nitrile groups in PAN, while in the second stage, the increased decomposition rate was due to the degradation of PS. Typically, PS begins to degrade between 330 and 380 °C. Around 450 °C, PS molecular chains depolymerize, producing monomers and oligomers that volatilize, leading to thermal loss. For ASM-14, weight loss mainly occurred between 290 and 460 °C. Throughout this range, the thermal decomposition rate was steady without significant fluctuations. When the temperature reached 800 °C, the final carbon yield for ASM-1, ASM-3, ASM-6, and ASM-14 were 27.79%, 33.89%, 30.31%, and 31.45%, respectively. This suggests that increasing the PS content can improve the residual mass retention, benefiting the thermal stability of the PAN/PS/PMMA fiber membrane. After incorporating PMMA into the blend, the carbon yield decreased, as PMMA completely decomposed at this temperature [50].

The thermal effects of the PAN/PS/PMMA fiber membrane and commercial Celgard separator were investigated in the temperature range of 20–800 °C. The DSC curves are shown in Figure 8b. The Celgard 2400 separator exhibited an endothermic peak at 168.25 °C, indicating its melting point. In contrast, the exothermic peaks of ASM-1, ASM-3, ASM-6, and ASM-14 appeared at 309.5, 307.8, 309.2, and 308.8 °C, respectively. A sharp exothermic peak was observed at 309.5 °C for ASM-1, attributed to the cyclization reaction of PAN. After blending with PS, the exothermic peak temperatures of ASM-3 and ASM-6 showed no significant shift; however, the peak intensities decrease noticeably—from 6.44 mW/mg(ASM-1) to 4.6 mW/mg (ASM-3) and 4.13 mW/mg (ASM-6). This indicates that the blending modification effectively regulates the cyclization reaction of PAN, resulting in a more gradual and uniform release of heat. With the further incorporation of PMMA, the exothermic peak intensity was further reduced, decreasing from 4.6 mW/mg (ASM-3) to 4.36 mW/mg (ASM-14). This suggests that the incorporation of PS and PMMA effectively delays the thermal decomposition process of the material, thereby reducing the risk of membrane failure under extreme temperature conditions and potentially providing critical time to interrupt thermal runaway in batteries. Furthermore, the absence of new exothermic peaks in the blended fibrous membranes confirms the good compatibility among PAN, PS, and PMMA.

In summary, compared with the Celgard 2400 membrane, ASM-14 exhibited a lower thermal shrinkage ratio, which helped to reduce the risk of internal short circuits caused by thermal contraction. It also showed a higher thermal transition temperature, indicating that ASM-14 can maintain structural integrity at elevated temperatures and delay the onset of thermal runaway chain reactions. Furthermore, the membrane demonstrated a slower thermal degradation rate, resulting in more uniform energy release at high temperatures and preventing violent reactions caused by localized overheating. In addition, its higher carbon yield suggests that the resulting carbonaceous residue may form an insulating layer that blocks short-circuit current pathways and reduces local heat accumulation.

### 3.4. Electrochemical Performance of the Battery

**Figure 9:**
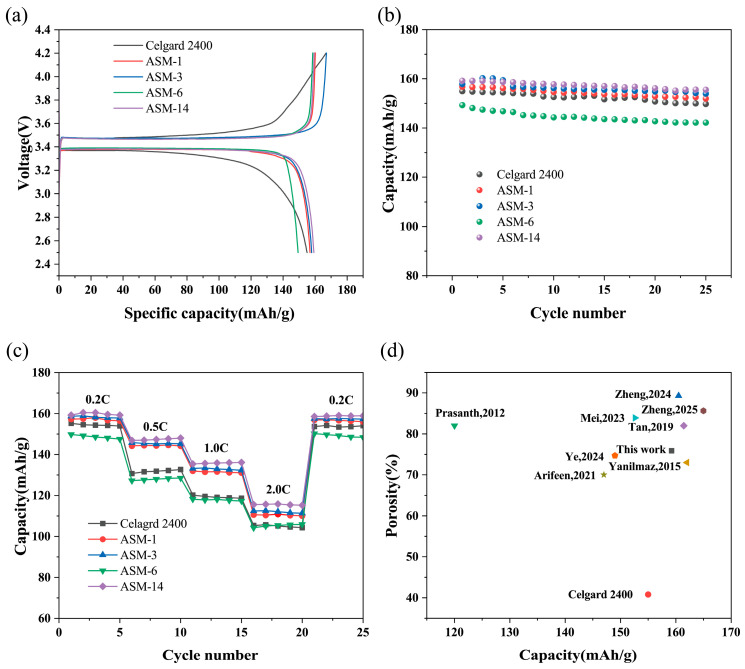
Electrochemical performance of batteries assembled with the separators and comparison with other reported studies: (**a**) Initial charge–discharge performance; (**b**) cycling stability; (**c**) rate performance; (**d**) performance comparison between this study and previously reported works [41,42,45,50,52,53,54,55].

The membranes prepared in this study were used to assemble batteries, and the performance of the lithium-ion batteries was evaluated through charge-discharge tests at a rate of 0.2C for 25 cycles. Figure 9a shows the first charge-discharge performance of the batteries. The first discharge capacity of the commercial separator was 155 mAh/g. The first discharge capacities of ASM-1, ASM-3, ASM-6, and ASM-14 were 157, 158, 149 and 159 mAh/g, respectively. Figure 9b illustrates the discharge capacity retention after 25 charge discharge cycles. After 25 cycles, the discharge capacity of the commercial Celgard separator was 150 mAh/g, with a capacity retention rate of 96.77%. The cycle discharge capacities of ASM-1, ASM-3, ASM-6, and ASM-14 were 152, 154, 142, and 156 mAh/g, with capacity retention rates of 96.82%, 97.47%, 95.3%, and 98.11%, respectively. ASM-14 exhibited excellent first-cycle charge-discharge performance and cycling stability, which can be attributed to its good electrolyte affinity and abundant porosity, helping retain more electrolyte and thus enhancing battery performance. 

The charge–discharge performance of the fiber membranes under high current rates was evaluated. As shown in Figure 9c, the rate performance test revealed that the battery assembled with ASM-14 delivered discharge capacities of 160, 148, 136, and 116 mAh/g at current rates of 0.2, 0.5, 1.0, and 2.0 C, respectively. These values were higher than those of the Celgard separator (155, 133, 120, and 106 mAh/g), ASM-1 (157, 145, 132, and 111 mAh/g), ASM-3 (159, 146, 133, and 113 mAh/g), and ASM-6 (150, 129, 118, and 106 mAh/g). This enhanced rate performance is attributed to the incorporation of PMMA in ASM-14, which exhibited good compatibility with lithium and a strong affinity toward the electrolyte, thereby promoting greater electrolyte retention and improving battery performance under high current densities.

### 3.5. Comparison of Overall Separator Performance

The key performance metrics of the separators investigated in this study are summarized and compared in Table 3. The results indicate that ASM-14 exhibited a moderate fiber diameter and achieved porosity and electrolyte uptake levels second only to ASM-1. It possessed the highest tensile strength among all prepared separators, with a thermal shrinkage rate below 3% and the slowest decomposition rate under high-temperature conditions. In the battery assembly tests, ASM-14 demonstrated the best performance in terms of initial charge–discharge capacity, cycling stability, and rate capability.

In summary, ASM-14 was identified as the ternary blended fiber membrane with the most balanced and superior overall performance in this study. It was prepared using a spinning solution concentration of 18 wt% with a composition ratio of PAN/PS/PMMA = 8:2:2.

The optimal PAN/PS/PMMA ternary blended fibrous membrane (ASM-14) prepared in this study was compared with membranes fabricated from similar materials using the centrifugal spinning (CS) and electrospinning (ES) methods reported in the literature, as illustrated in Figure 9d and summarized in Table 4. The results showed that the PAN/PS/PMMA membrane produced via centrifugal spinning exhibited comparable porosity and superior tensile strength relative to the other membranes. Furthermore, under identical current rates, the battery assembled with the PAN/PS/PMMA (ASM-14) membrane demonstrated discharge capacities comparable to, or even better than, those of the other reported membranes.

## 4. Conclusions

In this study, PAN/PS/PMMA ternary blended fibrous membranes were successfully fabricated using centrifugal spinning, combining the advantages of PAN, PS, and PMMA. When the PAN/PS/PMMA ratio was 8:2:2 with a total concentration of 18 wt% (denoted as ASM-14), the resulting fibrous membrane exhibited excellent thermal stability, with a thermal shrinkage rate of less than 3% after being heated at 150 °C for 1 h. The TGA results confirmed the uniformity of the exothermic process, while DSC analysis revealed a reduction in the peak temperature of the cyclization exotherm. The membrane also demonstrated favorable porosity, electrolyte uptake, and hydrophilicity. Electrochemical testing revealed that lithium-ion batteries employing the fabricated separator exhibited an initial discharge capacity of 159 mAh/g at a current rate of 0.2 C, with a capacity retention of 98.11% after 25 cycles, demonstrating great cycling stability. Even at higher current rates of 0.5, 1.0, and 2.0 C, the batteries maintained high discharge capacities of 148, 136, and 116 mAh/g, respectively. Therefore, the PAN/PS/PMMA ternary blended fibrous membrane (ASM-14) prepared via centrifugal spinning exhibits potential for practical application in lithium-ion batteries.

## Figures and Tables

**Table 1:** Preparation schemes for PAN fiber membranes with different mass fractions.

Number	PAN Solution Mass Fraction (%)	Stirring Time (h)/Temperature (°C)	Needle Inner Diameter (G)	Rotating Speed (r/min)
1	20	22/80	28	4500
2	20	9/80	28	4500
3	20	9/80	26	4500
4	20	9/80	24	4500
5	18	9/80	28	4500
6	18	9/80	26	4500
7	18	9/80	24	4500
8	16	9/80	28	4500
9	16	9/80	26	4500
10	16	9/80	24	4500

**Table 2:** Preparation schemes for PAN/PS/PMMA fiber membranes with different ratios.

Number	PAN/PS/PMMA Ratio	Stirring Time (h)/Temperature (°C)	Needle Inner Diameter (G)	Rotating Speed (r/min)
1	10:0:0	9/80	26	4500
2	9:1:0	9/80	26	4500
3	8:2:0	9/80	26	4500
4	8:2:0	9/80	28	4500
5	8:2:0	9/80	24	4500
6	7:3:0	9/80	26	4500
7	7:3:0	9/80	28	4500
8	7:3:0	9/80	24	4500
9	6:4:0	9/80	26	4500
10	8:2:0.5	9/80	26	4500
11	8:2:0.5	9/80	28	4500
12	8:2:1	9/80	26	4500
13	8:2:1	9/80	28	4500
14	8:2:2	9/80	26	4500
15	8:2:2	9/80	28	4500
16	8:2:3	9/80	26	4500
17	8:2:3	9/80	28	4500

**Table 3:** Performance comparison of the separators prepared in this study.

Sample	Celgard 2400	ASM-1	ASM-3	ASM-6	ASM-14
Fiber diameter distribution (nm)	-	500–800	2000–3000	3000–4000	2000–3000
Porosity (%)	40.82	81.27	68.23	64.21	75.87
Electrolyte uptake (%)	157	392	317	282	346
Contact angle (°)	112.2	53.3	99.6	103.5	79.3
Tensile strength (MPa)	163.16	7.52	11.44	20.96	23.48
Thermal shrinkage ratio (%)	28.9	0.28	0.12	3.18	2.85
Initial discharge capacity (mAh/g)	155	157	158	149	159
Capacity retention after cycling (mAh/g)	150	152	154	142	156
Capacity retention (%)	96.77	96.82	97.47	95.3	98.11
Discharge capacity at 2.0 C (mAh/g)	106	111	113	106	116

**Table 4:** Comparison of the performance of the membranes prepared in this study with those reported in the literature.

Separator Material	Porosity (%)	Tensile Strength (MPa)	Capacity (mAh/g)	Method/Efficiency	Ref.
Celgard 2400	40.82	125.17	155 (0.2 C)	/	/
PAN-PMMA	89.28	10.85	160.5 (0.2 C)	CS/100mL/h	[50]
PAN-PS-PMMA	80–84	/	120 (0.1 C)	ES/12mL/h	[52]
PAN-TPU-PS	82	10.8	161.44 (0.1 C)	ES/0.5mL/h	[41]
PMMA-PAN	73	/	162 (0.2 C)	CS	[42]
PU-PAN	83.9	/	152.6 (0.2 C)	CS/100mL/h	[53]
PAN-PVDF-PAN	85.64	11.03	165 (0.5 C)	ES/0.6mL/h	[54]
PAN-PI-SiO2	70	18.9	147 (1 C)	ES/1.8 mL/h	[55]
PVDF-PMMA	74.68	22.09	149 (0.2 C)	ES/1.4mL/h	[45]
PAN-PS-PMMA	75.87	23.48	159 (0.2 C)	CS/100mL/h	This work

## Data Availability

Due to limitations such as privacy or ethics, data are available upon request. The data presented in this study are available upon request from the corresponding authors. Due to privacy issues involved in the laboratory and the team’s testing process, the data are not made public.
